# Patient questionnaire following closure of tracheotomy fistula: percutaneous vs. surgical approaches

**DOI:** 10.1186/2052-0492-2-17

**Published:** 2014-02-24

**Authors:** Yukihiro Ikegami, Ken Iseki, Chiaki Nemoto, Yasuhiko Tsukada, Jiro Shimada, Choichiro Tase

**Affiliations:** Department of Emergency and Critical Care Medicine, School of Medicine, Fukushima Medical University, 1 Hikarigaoka, Fukushima, 960-1295 Japan

**Keywords:** Percutaneous tracheotomy, Long-term outcome, Questionnaire, Door-to-door evaluation

## Abstract

**Background:**

Tracheotomy is an indispensable component in intensive care management. Doctors in charge of the intensive care unit (ICU) usually decide whether tracheotomy should be performed. However, long-term follow-up of a closed fistula by these doctors is rarely continued in most cases. Doctors in charge of the ICU should be interested in the long-term prognosis of tracheotomy. The purpose of this study was to evaluate whether different tracheotomy procedures affect the long-term outcome of a closed tracheal fistula.

**Methods:**

We mailed questionnaires to patients undergoing tracheotomy in Fukushima Medical University Hospital between January 2008 and December 2010. Questions concerned problems related to perception, laryngeal function, and the appearance of a closed fistula. Patients were classified into percutaneous tracheotomy (PT) group and surgical tracheotomy (ST) group. We evaluated the statistical significance of differences in the frequency and degree of each problem between the two groups. A door-to-door objective evaluation using the original scoring system was then performed for patients who replied to the mailed questionnaire. We evaluated the percentage of patients with high scores as well as the mean scores for problems with function and appearance.

**Results:**

We received completed questionnaires from 28/40 patients in the PT group and 35/55 patients in the ST group. There were no significant differences in age, mean hospital stay, or APACHE II score between the groups. Regarding problems with appearance, the outcomes of PT were significantly better than those of ST with respect to self-evaluation (*p* = 0.04) and the frequency (*p* = 0.03) and degree (*p* = 0.02) of scar unevenness according to door-to-door evaluation. However, there were no significant differences in the frequency or degree of self-evaluation in problems with perception and function between the two groups. There were no significant differences in the frequency or degree of door-to-door evaluation of problems with function.

**Conclusions:**

This study shows that PT might be superior to ST with respect to problems with long-term appearance. Continuous follow-up of closed tracheal fistulas can help assure that patients recovering from a critical condition experience a better return to their former lives. A systematic follow-up of post-critical-care patients is required.

## Background

In cases of prolonged mechanical ventilation (MV), tracheotomy is taken into consideration by the doctors in charge of the intensive care unit (ICU). Doctors continue intensive care after tracheotomy and are satisfied with the accomplishment of difficult respiratory management when patients can be successfully weaned from MV. However, most doctors have little clinical interest in patients once they leave the ICU after weaning from MV. These doctors are responsible for the decision to perform tracheotomy, but most of them do not follow-up on the long-term condition of patients following closure of a tracheal fistula. In many cases, patients have to return to their former daily life, but they may have serious problems after tracheotomy.

There are currently two widely used tracheotomy methods, namely, percutaneous tracheotomy (PT) [1, 2] and conventional surgical tracheotomy (ST). Both methods have advantages and disadvantages, and at present, there are no adaptive criteria for selection. Doctors in charge of the ICU usually select a tracheotomy method according to the patient's clinical condition or anatomical features of the neck. In Fukushima Medical University Hospital, PT is used as often as possible because it can be performed by the emergency staff at the bedside. This is important because emergency doctors are in charge of the ICUs in our hospital.

The purpose of this study was to evaluate whether different tracheotomy procedures affect the long-term outcome of a closed tracheal fistula. Our goal is to provide emergency medical care that assures a positive return to patients' former lives.

## Methods

We selected electronic medical records of all patients who had undergone tracheotomy in the emergency department of Fukushima Medical University Hospital between January 2008 and December 2010. We mailed survey questionnaires to surviving patients with closed tracheal fistulas. For this study, we regarded the patients as materials sufficiently if it took more than 6 months after removal of a cannula, in accordance with Ciaglia and Graniero's study [3], which reported long-term follow-up (4 months after percutaneous tracheotomy in short cases). The replies of patients who received continuous treatment following their tracheotomy were excluded from the study analysis. In addition, patients who were extremely unstable and patients with conditions likely to influence the long-term prognosis after tracheotomy were not included. The perioperative clinical courses of all cooperating patients were investigated in detail from the operative records in the electronic charts. Experienced emergency specialists performed all of the PT procedures at bedside in the ICU using the Neo Perc™ (Covidien Group, Tokyo, Japan). Otolaryngology specialists performed all of the ST procedures in the operating room using standard methods.

The contents of the questionnaire were based on self-evaluation of the problems after tracheotomy. In this study, problems were classified into problems with perception, function, or appearance. We showed patients examples of each symptom, and the patients freely wrote about their problems by self-evaluation. We established a scale so that the patients might perform a self-evaluation of the degree of each problem. The scale ranged from 0 to 10 points, and severe problems were assigned a high score (0 points, none; 10 points, strong and continuous). All valid replies were classified into either the PT or ST group. The frequency of each type of problem (classified into the three categories of perception, function, and appearance) and the degree of each problem as evaluated by the self-scoring system were compared between groups. If one patient declared several concerns falling under one type of problem, we totaled each separately. The mean value of each score was independently compared between the two groups.

Next, we performed a door-to-door objective evaluation by interviewing patients who replied to the questionnaires. We established a scoring system for evaluation of problems with function and appearance. Functional problems included dysphagia (3 points, severe dysphagia; 2 points, slight dysphagia; and 1 point, no dysphagia) and dysphonia (3 points, severely hoarse voice; 2 points, slightly hoarse voice; and 1 point, no hoarse voice). Appearance problems included size (3 points, scar length of ≥1.5 cm; 2 points, scar length of ≥1 and <1.5 cm; and 1 point, scar length of <1 cm), unevenness (3 points, depressed or prominent scar of ≥5 mm; 2 points, depressed or prominent scar of <5 mm; and 1 point, no depressed or prominent scar), and pigmentation (3 points, bright color; 2 points, light color; and 1 point, no pigmentation). In the door-to-door objective evaluations, we compared not only the percentage of patients whose score was above 2 points, but also the mean score itself.

We used unpaired *t* tests for continuous data and chi-square tests for categorical data, except when expected cells were less than 5, in which case we used Fisher's exact test. We used IBM SPSS software version 21 (SPSS, Chicago, IL, USA) for the statistical calculations. All of the tests were two-tailed, with significance set at *p* < 0.05. This study was approved by the ethics committee of Fukushima Medical University, and we strictly followed our institutional guidelines for the protection of personal data. We obtained written informed consent for data disclosure and study participation from all study participants.

## Results

Patient demographics are shown in Table 1. We received 28 replies from 40 PT patients (70.0%) and 35 replies from 55 ST patients (63.6%). There were no significant differences in mean age (*p* = 0.56), mean hospital stay (*p* = 0.10), Acute Physiology and Chronic Health Evaluation II (APACHE II) score (*p* = 0.79), or the rate of return to former life (*p* = 0.52) between the groups. There was a tendency for a high number of diagnoses of exogenous diseases in the PT group. Cardiovascular disease was significantly more frequent in the ST group (*p* < 0.05). The mean time to tracheotomy in the PT group was significantly shorter than that in the ST group (*p* < 0.05). The mean time of removal of the tracheal cannula was shorter in the PT group than in the ST group (*p* < 0.05). There was no significant difference in the mean time since removal of the tracheal cannula between the two groups (*p* = 0.64). There were no recorded perioperative complications in either group. With respect to reasons for tracheotomy, PT was significantly more frequently performed in patients in whom prolonged MV was anticipated (*p* < 0.05). In contrast, ST was significantly more frequently performed in patients who underwent prolonged MV (*p* < 0.05).

**Table 1:** **Patient demographics**

Respondents	PT (28/40)	ST (35/55cpa)	Significance
Response rate (%)	70.0%	63.6%	NS
Male/female	18/10	20/15	NS
Mean age ± SD	62.1 ± 19.3	62.9 ± 20.8	NS
Mean hospital day ± SD	38.0 ± 10.2	41.2 ± 12.1	NS
APACHE II score ± SD	24.0 ± 10.5	24.3 ± 8.8	NS
Return to former life (%)	24 (85.8%)	28 (82.9%)	NS
Clinical diagnosis			
Multiple trauma	7	5	NS
Acute intoxication	5	4	NS
Burn	3	2	NS
Sepsis	4	5	NS
Heat stroke	1		
Meningitis	1		
Cardiovascular disease	4	12	*p* < 0.05
Acute pneumonia	3	5	NS
Acute pancreatitis		1	
Hyperglycemic acidosis		1	
Mean time to tracheotomy (mean days ± SD)	9.1 ± 2.9	14.5 ± 2.6	*p* < 0.05
Mean time to removal of tracheal cannula (mean days ± SD)	36.9 ± 22.1	54.7 ± 22.2	*p* < 0.05
Mean time since removal of tracheal cannula (mean days ± SD)	24.5 ± 19.8	21.5 ± 20.2	NS
Reasons for tracheotomy			
Anticipation of prolonged MV	14	9	*p* < 0.05
Prolonged MV more than 14 days (including failed cases of wearing from MV)	7	21	*p* < 0.05
Airway stenosis	7	5	NS

All test were two-tailed, with significance set at *p* < 0.05. *PT* percutaneous tracheotomy, *ST* surgical tracheotomy, *SD* standard deviation, *NS* not significant, *MV* mechanical ventilation.

**Figure 1:**
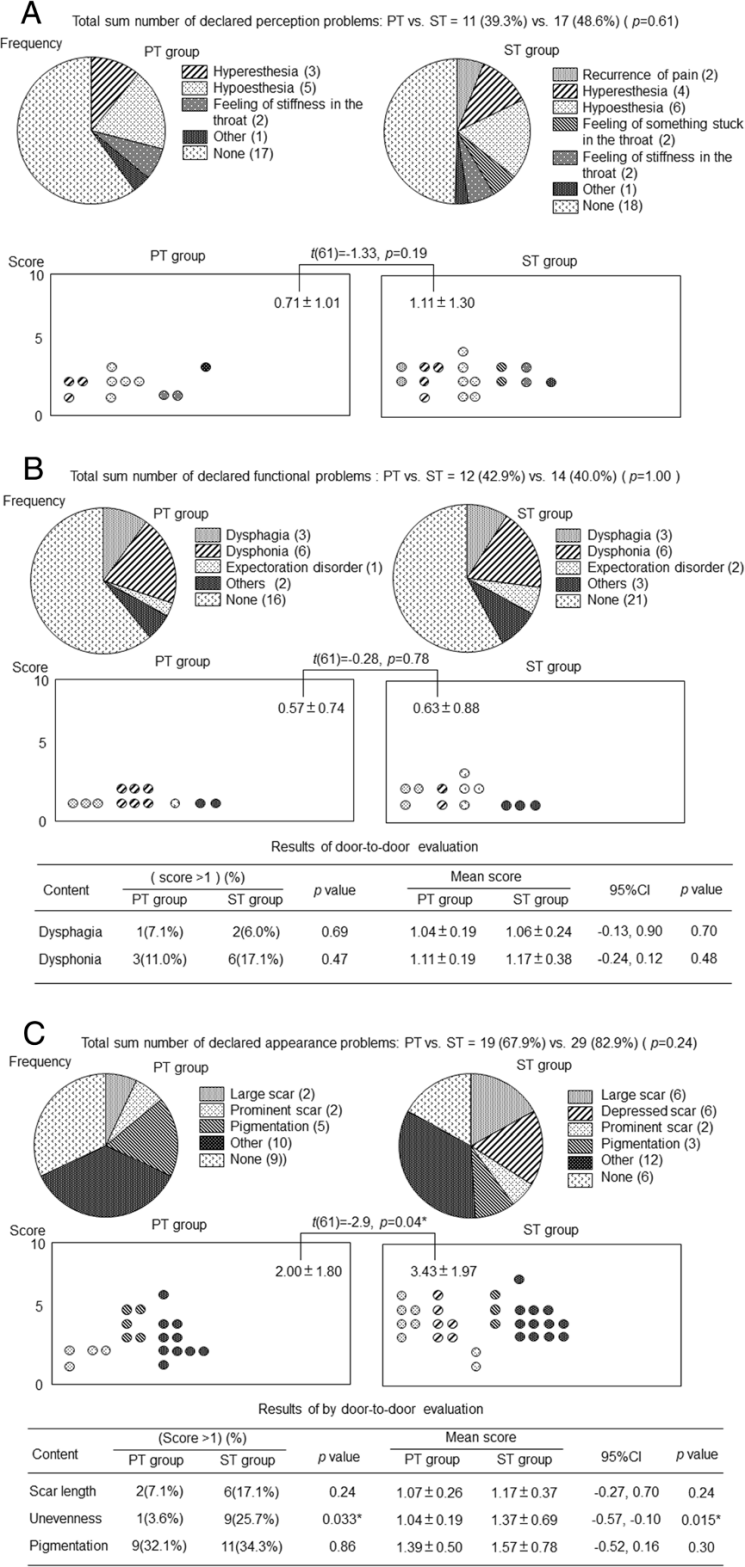
**Comparison of long-term outcomes with regard to different problems between the PT and ST groups. (A)** Perceptual problems. There were no significant differences between the PT and ST groups in the self-evaluated frequency (11/39 vs. 17/35, respectively, *p* = 0.61) or mean score of the degree of perceptual problems (0.71 ± 1.01 vs. 1.11 ± 1.30, respectively, *p* = 0.19). **(B)** Functional problems. There were no significant differences in the self-evaluated frequency (*p* = 1.00) or score of the degree (*p* = 0.78) of functional problems between the two groups. There were no significant differences in the frequency of a high score (score ≥2 points) or in the mean score for dysphagia and dysphonia in the door-to-door evaluation. **(C)** Appearance problems. There was no significant difference in the frequency of appearance-related problems between the PT and ST groups (19/28 vs. 29/35, respectively, *p* = 0.24). However, the mean score for the degree of appearance-related problems was significantly higher in the ST group than in the PT group (3.43 ± 1.97 vs. 2.00 ± 1.80, respectively, *p* = 0.04). There were no significant differences in the frequency of a high score (score ≥2 points) or in the mean score for scar length and pigmentation in the door-to-door evaluation. However, unevenness of >2 points was significantly more frequent in the ST group than in the PT group (9/35 vs. 1/28, respectively, *p* = 0.03).

Outcomes of perceptual problems are shown in Figure 1A. Two patients in the ST group reported sudden and repeated stabbing pain but stated that it was mild and of short duration. One patient in each group reported feeling slight discomfort that was difficult to define or explain (categorized as ‘other’ , Figure 1A). Although these symptoms were vague and the validity of their inclusion is debatable, we included them because of appeals made by the patients. There were no significant differences in the self-evaluated frequency (11/39 vs. 17/35, *p* = 0.61) or mean score of the degree of problems (0.71 ± 1.01 vs. 1.11 ± 1.30, *p* = 0.19) between the two groups.

The outcomes of functional problems are shown in Figure 1B. Two patients in the PT group and three patients in the ST group reported that their throat function had deteriorated slightly, with no significant differences in the self-evaluated frequency (*p* = 1.00) or score of the degree (*p* = 0.78) of these problems between the two groups. In the door-to-door evaluation, there were no significant differences in either frequency of a high score (≥2 points) or the mean scores for dysphagia and dysphonia. We observed no cases of severe dysphagia in either group. Several patients in both groups reported voice changes, but we observed no severely hoarse voices during the interviews.

The outcomes of appearance problems are shown in Figure 1C. Ten patients in the PT group and fourteen in the ST group stated that they did not expose the scar despite the fact that it was barely visible. There was no significant difference in the frequency of problems with appearance between the two groups (19/28 vs. 29/35, *p* = 0.24). However, the mean score of the degree of problems was significantly higher in the ST group than in the PT group (3.43 ± 1.97 vs. 2.00 ± 1.80, respectively, *p* = 0.04). In the door-to-door evaluation, there were no significant differences in either the frequency of high scores (≥2 points) or the mean scores for scar length and pigmentation. However, unevenness of >2 points was significantly more frequent in the ST group than in the PT group (9/35 vs. 1/28, *p* = 0.03). In addition, the mean score of the degree of scar unevenness in the ST group was significantly higher than that in the PT group (1.37 ± 0.69 vs. 1.04 ± 0.19, respectively, *p* = 0.02).

## Discussion

Respiratory care using MV is indispensable for patients with critically ill conditions, such as multiple traumas or severe burns. When prolonged MV is anticipated, doctors in charge of the ICU must consider tracheotomy. Doctors in charge of the ICU perform tracheotomy whenever necessary, but most of them are not overly concerned about how the fistula recovers after removal of the tracheal cannula and do not make the effort to determine the resulting problems that patients experience. These doctors should be more interested in the daily life of patients and post-critical care. Tracheotomy is invasive, with a resulting scar on the front of the neck. Therefore, a method that can be performed with minimal invasion should be selected.

Presently, there are two methods of tracheotomy, namely, PT and ST. PT is a technique that was first reported by Ciagria et al. in 1985 [2]. Since then, its use has spread worldwide, and it has undergone several modifications over the last 20 years [4]. PT is simple and can be performed at the bedside by trained intensivists [5–7]. Several other authors have reported long-term outcomes of PT [8–11]. However, there are no studies based on scores obtained by patients' self-evaluations, such as our design in the current study. Although a patient's self-evaluation might not be objective, we recognized that talking to patients is important and thus designed our method by questionnaire. We hypothesized that the outcomes for all of the problems with long-term perception, function, and appearance in the PT group are superior to those in the ST group. However, there were no significant differences in the frequency and degree of problems with perception and function in self-evaluation between the two groups. In addition, in the door-to-door evaluation, there were no significant differences in problems with function between the two groups.

We found significant differences in the score of the degree of problems with appearance in the self-evaluation and in the score of scar unevenness in the door-to-door evaluation. PT was superior to ST in these evaluations. These data suggest that PT is more effective than ST with respect to the long-term appearance of a closed tracheal fistula. Several factors that promote good recovery of a fistula following PT are as follows: (1) this procedure can be performed with a small incision; (2) PT does not require any separation of subcutaneous tissue, including the sternohyoid; and (3) infection can be reduced because the procedure does not require vessel ligation and because the primary tracheal fistula created by a dilator is small, preventing the leakage of tracheal secretions into the wound. We recommend considering PT for an optimal long-term appearance.

**Figure 2:**
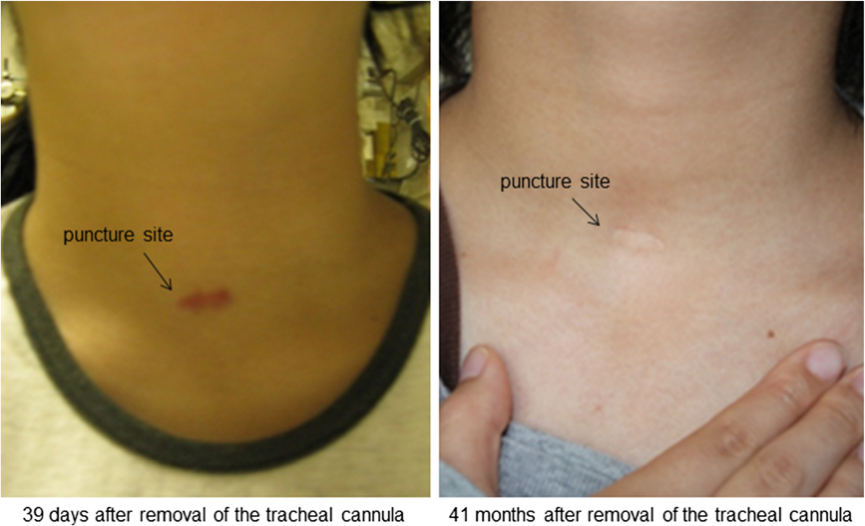
**Photographs of one patient's tracheotomy scar.** The patient is an 18-year-old woman. The appearance of the closed tracheal fistula was judged to be very good in the door-to-door evaluation, but the patient commented that she always conceals the scar by wearing collared clothes when going out in public.

Of the patients who replied to our questionnaire, approximately 40% reported that they had some problems with perception and function by self-evaluation in both groups. For appearance, the majority of patients in both groups reported that they had some problems as assessed by self-evaluation. However, only a few patients pointed out functional problems in the door-to-door evaluation. In addition, problems with appearance widely varied between the self-evaluation and door-to-door objective evaluation. An example of these variations was seen when, following receipt of the patients' completed questionnaires, we visited the home of an 18-year-old woman who had undergone PT because of intractable epileptic seizures. We observed the appearance of the patient's closed tracheal fistula and judged its condition as good. However, the patient stated that she always concealed the scar by wearing clothes with a collar when in public (Figure 2). Similarly, a 67-year-old woman who had undergone ST because of severe heart failure was also concerned about the effects of tracheotomy on her appearance and hoped that plastic surgery would be able to remove the scar. The dissatisfaction level after tracheotomy might be greater than we assumed, and it might not be related to the method used. Several authors have reported the potential merits of early tracheotomy [12–17], and we expected that early tracheotomy would be acceptable if PT provides a good long-term prognosis of a closed fistula. However, our study results were not consistent with this expectation as evidenced by the patients' long-term dissatisfaction. Medical staffs are likely to regard the merits of simplicity in respiratory management by tracheotomy, but perioperative assessment regarding the indication or timing before tracheotomy should be performed. Furthermore, the long-term prognosis of a closed fistula should be explained in detail.

Our study has several limitations. First, our standard for door-to-door evaluation was established specifically for this study. However, the number of patients included in the study was small, and we need to collect more cases to verify the validity of this standard. We were unable to show any significant differences in the long-term prognosis with regard to perceptual and functional problems between the PT and ST groups. Second, the variation in diseases between the two groups might be an important factor contributing to the significant difference in the mean time to tracheotomy or mean time of cannulation. Third, we consider a patient's self-evaluation to be important in evaluating his or her long-term prognosis. However, more invasive assessment by an otolaryngologist, such as laryngeal fiberscopy, may be necessary for a more objective evaluation. Because the performance of PT or ST made a difference, the significant difference was brought at the time to performance between the two groups. Therefore, a randomized and controlled prospective study should be conducted to precisely evaluate the long-term prognosis of PT and ST. We would like to establish a long-term follow-up system for tracheotomy based on the findings of this study.

## Conclusion

This study shows that PT might be superior to ST for problems with long-term appearance. However, the sample size of our study was small and there were differences in the diseases between the PT and ST groups. Continuous follow-up of closed tracheal fistulas can help assure that patients recovering from a critical condition experience a better return to their former lives. A systematic follow-up of post-critical-care patients is required.

## Abbreviations

ICU: intensive care unit
MV: mechanical ventilation
PT: percutaneous tracheotomy
ST: surgical tracheotomy.
